# An analysis of the “Half-Perc” versus open surgical placement method for a peritoneal dialysis catheter: a non-inferiority cohort study

**DOI:** 10.1186/s12882-020-01936-0

**Published:** 2020-07-20

**Authors:** Difei Zhang, Yu Peng, Tingting Zheng, Hui Liu, Jianfeng Wu, Zewen Li, Jingxu Su, Yuan Xu, Xiaoxuan Hu, Guowei Chen, Haijing Hou, La Zhang, Liwen Wu, Xusheng Liu, Fuhua Lu

**Affiliations:** 1 The Second Clinical College of Guangzhou University of Chinese Medicine, No.111 Dade Road, Guangzhou, 510405 China; 2 Department of Nephrology, Guangdong Provincial Hospital of Chinese Medicine, No.111 Dade Road, Guangzhou, 510120 China

**Keywords:** Half-Perc, Open surgery, Peritoneal dialysis, Catheter placement, Clinical outcome

## Abstract

**Background:**

Most end-stage renal disease (ESRD) patients undergo open surgical techniques for peritoneal dialysis (PD) catheter placement. An alternative method to PD catheter implantation is the half-percutaneous (“Half-Perc”) technique based on a modified trocar that is performed by a nephrologist. The single-center, retrospective, observational, cohort study presented here aimed to compare the effects of the “Half-Perc” technique with the traditional open surgery on peritoneal catheter insertion.

**Methods:**

From January 2015 to January 2018, 240 ESRD patients who received initial PD catheter placement were divided into two groups based on the “Half-Perc” technique or open surgery. All patients were followed up for 365 days or until loss of initial PD catheter or death. Prism 5 software was used to analyze baseline characteristics, operation-related parameters, mechanical complications and clinical outcomes.

**Results:**

The “Half-Perc” technique showed shorter operation time, shorter incision length, lower postoperative pain scores and quick initiation of the PD program compared to the open surgery. After the 365-day follow-up, the “Half-Perc” group showed a higher rate of catheter dysfunction (4% versus 0.9%) that was corrected by conservative treatment in most patients and a lower rate of peritonitis (4% versus 9.6%) but mechanical complications and clinical outcomes did not differ between the two groups. There was also no significant difference based on overall patient mortality or catheter removal. One-year initial catheter survival and true catheter survival were not statistically different between the groups.

**Conclusion:**

The “Half-Perc” placement of the PD catheter using a modified metal trocar appears to be a non-inferior alternative method and carries minimal invasiveness and risk compared to open surgical placement.

## Background

Peritoneal dialysis (PD) is a well-accepted modality of dialysis therapy for patients diagnosed with end-stage renal disease (ESRD). The main advantages of PD include improved mobility, lower costs, less dietary restrictions and better preservation of residual renal function compared to haemodialysis (HD) [1]. The success of PD depends on timely, durable and functioning PD catheter access [2]. The PD catheter placement technique has evolved from a traditional open surgery, percutaneous technique to a surgical laparoscopy technique [3, 4]. However, there is still ongoing debate regarding the ideal technique for peritoneal catheter insertion.

Over the past few decades, the most commonly used technique for PD catheter placement is traditional open surgery, which is characterized by its simplicity, low costs, and lack of need for advanced equipment [5]. However, this is often associated with higher morbidity due to mechanical trauma and postoperative catheter malposition. In recent years, the peritoneoscopic method [6] and laparoscopic approaches for PD catheter implantation have been widely accepted with several benefits of accurate positioning of the peritoneal cavity [7]. However, these methods require general anesthesia, special equipment, advanced technique and cost are pricey. The percutaneous technique using the trocar or guide wire (Seldinger technique), is considered as simple and minimally invasive, but may cause inadvertent injury to the bowel and result in dialysate leakage [8]. In addition, percutaneous methods are not generally suitable for at-risk ESRD patients, particularly those with a history of abdominal surgery [9]. Thus, definitive evidence-based recommendations for the optimal insertion technique are difficult to surmise. A half-percutaneous (“Half-Perc”) technique based on a modified metal trocar in China is an alternative method of PD catheter implantation performed by nephrologists [10]. This novel technique was first introduced by Zhu Bai et al. in 2002 [11, 12] and shares some of the advantages of the percutaneous approach for PD catheter placement [13], including shorter operation duration and generally minimal tissue trauma, which results in less pain and a quick recovery [14].

Since the “Half-Perc” technique was performed in our PD center in 2015, there has been indication of some benefits related to clinical efficacy. The major objective of the cohort study presented here was to test whether patients receiving PD catheters through the “Half-Perc” insertion technique have clinically non-inferior outcomes compared to those receiving catheters through the open surgical insertion technique.

## Methods

### Patients

**Fig. 1:**
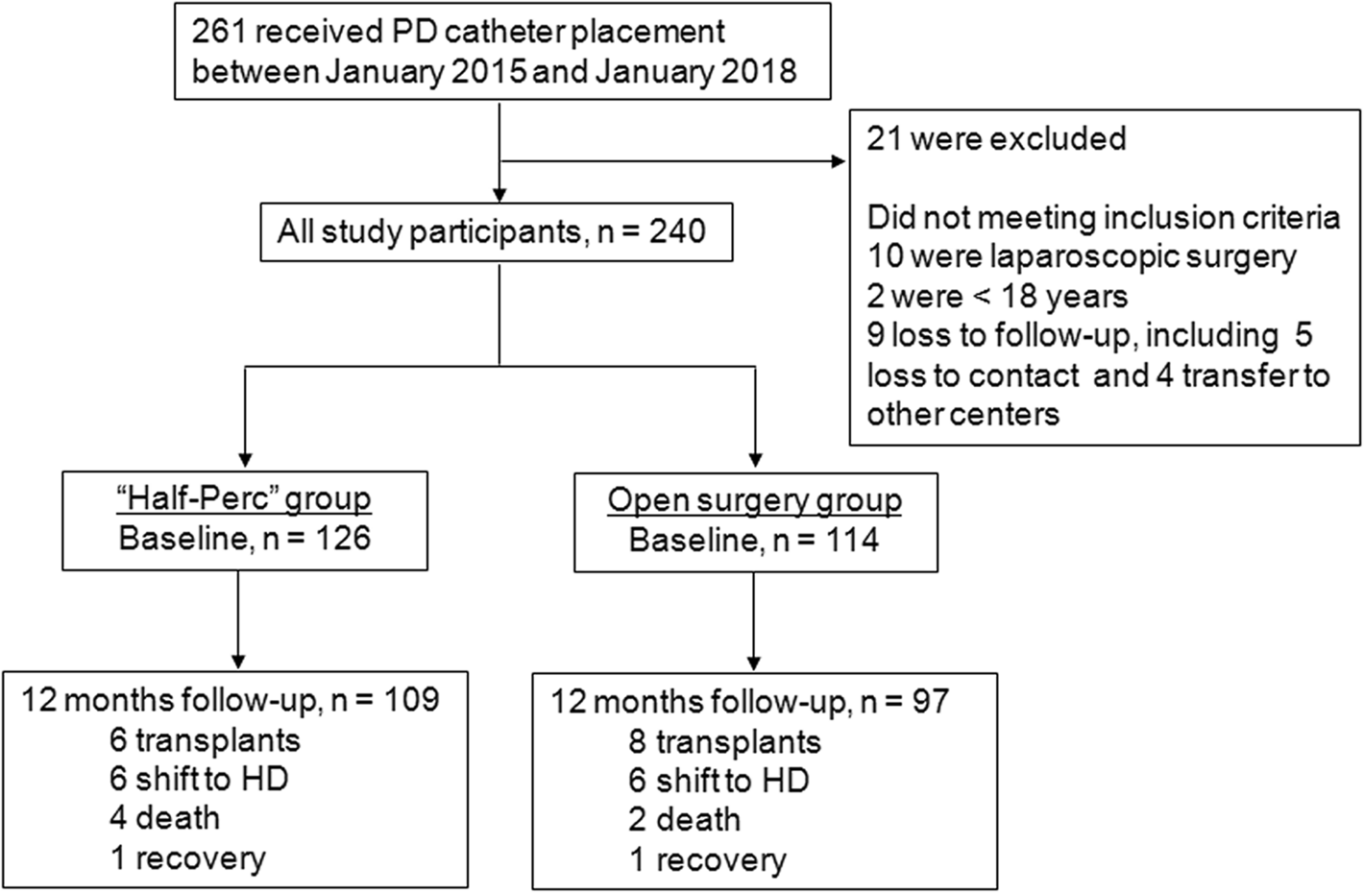
Schematic of the study flow chart

The study enrollment period was from 1 January 2015 to 31 January 2018 and follow-ups were completed by 31 January 2019. During this period, 261 ESRD patients underwent first PD catheter placement and were treated with PD at the Guangdong Provincial Hospital of Chinese Medicine. We excluded 21 patients from this analysis since they did not meet certain inclusion criteria: 10 patients underwent laparoscopic surgery, 2 patients were < 18 years of age and 9 patients did not have records of follow-up. The remaining 240 patients were included and analyzed further. Figure 1 represents the flow chart used for this study. All patients provided informed consent and this study was approved by the Institutional Ethics Review Boards of Guangdong Provincial Hospital of Chinese Medicine.

### Data collection

Patient information was collected manually by reviewing hospital electronic medical record. Baseline demographics, disease characteristics, operation-related parameters and mechanical complications and clinical outcomes were compared between the two study groups. All patients were followed for 1-year after catheter insertion or until loss of initial function of the catheter or death.

Factors that were analyzed included: (1) baseline characteristics, such as sex, age, body mass index, mean arterial pressure, primary disease, history of previous abdominal surgery and laboratory indexes, (2) operation-related parameters, such as operative type, operative time, incision length, bleeding volume, postoperative pain, use of analgesics, success rate of surgery, operative costs and delay in PD start, (3) mechanical complications, such as catheter malfunction including catheter migration, dialysate leakage, persistent bleeding and hernia, (4) clinical outcomes, such as peritonitis, exit-site/tunnel infections, patient mortality, causes of catheter removal and initial/true catheter survival rate.

### Catheter placement technique

All surgical procedures were performed by the same group of three nephrologists in the operating room on patients who have received local anesthesia. These three experienced nephrologists, who are senior attending physician at our PD center, are familiar with traditional open surgical and the “Half-Perc” placement method. All patients used the standard straight Tenckhoff catheter in this study.

### The “half-Perc” technique

**Fig. 2:**
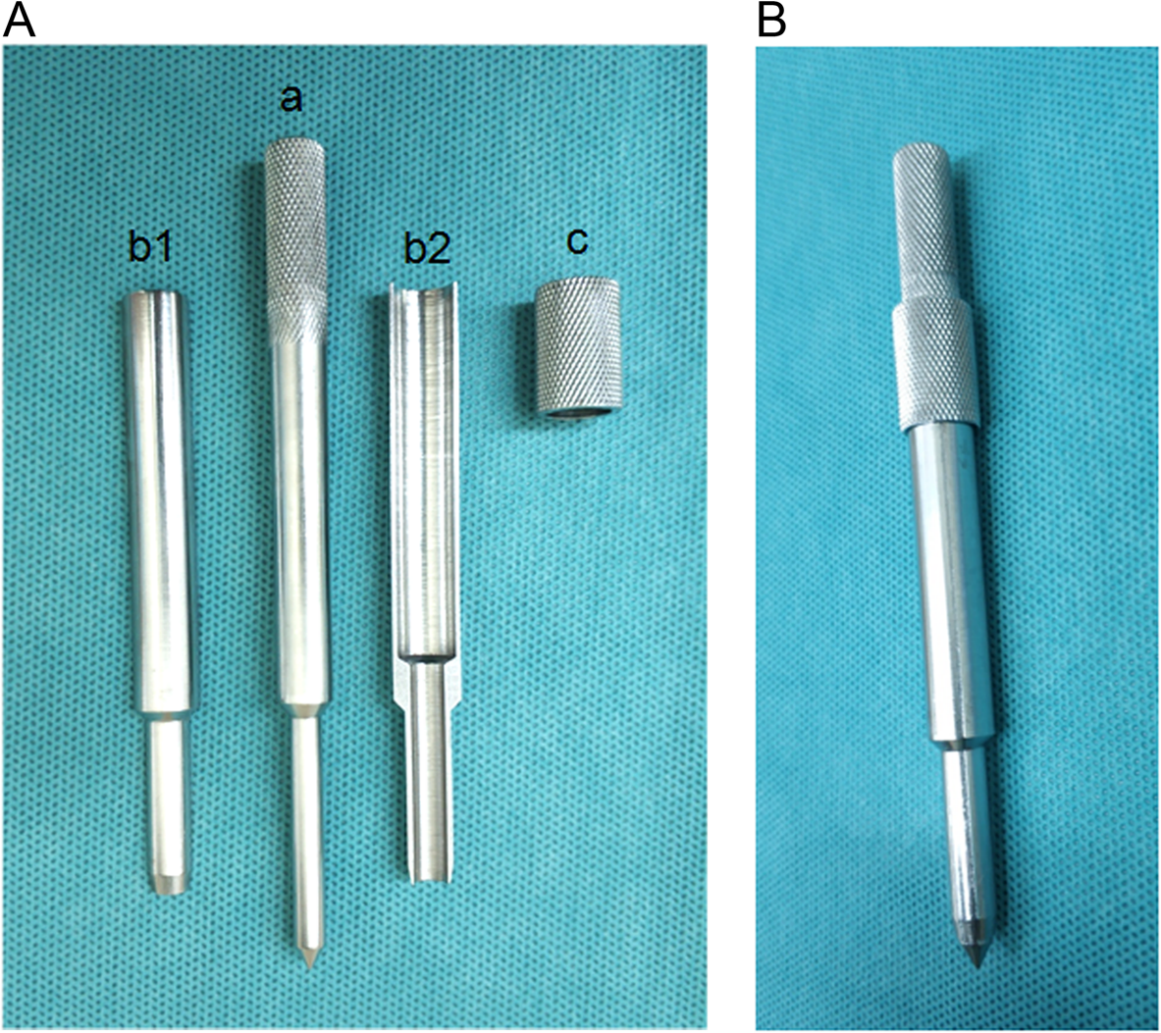
Components of the trocar device. A. The structure of a modified trocar: **a** Core-needle. **b** Metal trocar, including 2 independent parts. **c** Hoop. B. Configuration of the modified trocar

The “Half-Perc” technique of PD catheter placement was performed by nephrologists using a modified metal trocar (Fig. 2). Details of “Half-Perc” catheter insertion has been previously described [10]. Briefly, patients were placed in the supine position. The surgery procedure was performed under local anesthesia using 1% lidocaine. A 2 cm paramedian skin incision was made in the abdomen 2 cm inferior to the umbilicus. A small incision ~ 0.5 cm length was made in the anterior rectus sheath and a purse-string suture with a diameter of ~ 0.5 cm was placed in the anterior rectus sheath but not tightened. The rectus muscle was dissected bluntly using a straight hemostat and was then inserted by a modified metal trocar. Patients were asked to inflate their abdomen and the posterior rectus sheath and parietal peritoneum was punctured slowly using the tip of the trocar. At that time, an obvious empty sensation is felt. Next, the tip of the trocar was inserted by 1–2 mm into the peritoneal cavity. A guidewire was vertically inserted 2 cm in the intra-abdominal segment through the hollow trocar after the core needle was removed. The PD catheter was inserted into the peritoneal cavity ~ 5 cm using the metal guide wire. The guide wire was directed to the pelvic cavity and inclined on the abdominal wall at a 45° Angle. The PD catheter was slowly inserted into the Douglas pouch. The peritoneal cavity was rapidly injected with 150 ml of warm saline after the metal guidewire and trocar were removed and the solution was quickly aspirated, which confirmed correct positioning of the implanted catheter. The purse-string previously placed in the anterior layer of the rectus sheath was sutured to fix the peritoneal catheter and ensure that the inner cuff was implanted into the rectus muscle. A subcutaneous tunnel tract was made at the upper and outer edge of the primary incision and the outer cuff was implanted more than 2 cm from the exit site. The incision was surgically closed. A total of 2000 ml of PD solution was injected and released immediately after surgery. An urgent-start PD program could immediately be started if necessary.

### Open surgery procedure

The open surgery procedure has been previously described [15]. Briefly, the incision site was made either 2 cm lateral to the paramedian area or 10 cm above the pubic symphysis if necessary. A sagittal incision of 4–5 cm length was made in the skin and moved towards the anterior rectus fascia. The posterior layer of the rectus sheath was incised to expose the parietal peritoneum after the abdominal rectus muscle had been dissected using a straight hemostat. A purse-string suture with a diameter of ~ 0.5 cm was placed into the peritoneum, but not tightened. The peritoneal catheter was inserted through the opening wound and then threaded into the pelvic cavity using guidance from a guide wire. After removing the guidewire, the purse string in the peritoneum was sutured just below the inner cuff. The peritoneal cavity was injected with 200 ml of warm saline several times to observe whether the dialysate was easily aspirated and to verify the correct positioning of the implanted catheter. The anterior rectus sheath was then closed using interrupted sutures with the inner cuff fixing in the abdominal rectus muscle layer and the catheter going through the rectus sheath at the upper area of the incision. A subcutaneous tunnel tract was made with a downward exit site using a tunnel needle. The outer cuff was placed more than 2 cm from the exit site. The incision was then stitched. The regular PD program was usually initiated 7 days post operation.

### Statistical analyses

Normally distributed continuous variables were expressed as mean ± SD, qualitative data as median and interquartile range, and absolute numbers and percentages. Statistical comparisons between two groups were made using the Mann–Whitney test for continuous variables or by Pearson’s χ2 test for categorical variables.

The Kaplan–Meier method was used for the analysis of initial catheter survival rate and true catheter survival rate. Initial catheter survival was defined as a functional initial catheter before its loss for any reason. True catheter survival excluded patients with catheter dropout due to clearly unrelated causes such as renal transplantation, renal recovery or death from unrelated diseases. The Prism5 software (GraphPad Software Inc., La Jolla, CA) was used for statistical analyses. A value of *p* < 0.05 was considered statistically significant and all tests performed were two-tailed.

## Results

### Baseline characteristics

Table 1 contains a list of baseline demographic and disease characteristics for the two groups analyzed. This study enrolled 137 men and 103 women, with the average age being 51.8 ± 14.9 years. No significant differences were found for patient age, MAP, primary disease and history of abdominal surgery. However, the “Half-Perc” group showed a higher number of males and showed higher average BMI.

**Table 1:** Baseline demographic and disease characteristics

Patient characteristics	“Half-Perc”	Open surgery	*P* value
Patient (n)	126	114	
Sex (male/female)	84/42	53/61	0.0016
Age (years)	50.6±14.3	52.8±15.6	0.3273
Smoker	36 (28.6%)	25 (21.9%)	0.2380
Alcohol user	15 (11.9%)	9 (7.9%)	0.3011
BMI (kg/m2)	23.9±4.1	22.0±2.6	0.0008
MAP (mmHg)	113.0±16.4	110.0±15.8	0.3470
**Primary disease**			0.2720
Glomerulonephritis	71 (56.3%)	59 (51.8%)	
Diabetic nephropathy	40 (31.8%)	31 (27.2%)	
Obstructive nephropathy	6 (4.8%)	8 (7.0%)	
Other causes	9 (7.1%)	16 (14.0%)	
**History of abdominal surgery**	6	10	0.1883
Appendectomy	3 (2.4%)	2 (1.8%)	
Cesarean section	0 (0.0%)	3 (2.6%)	
Nephrectomy	1 (0.8%)	0 (0.0%)	
Others	2 (1.6%)	5 (4.4%)	
**Laboratory values**			
Serum creatinine (umol/L)	879.7±280.2	867.2±284.7	0.9273
Blood urea nitrogen (mmol/L)	27.3±11.3	26.5±12.6	0.5695
Serum albumin (g/L)	34.9±5.0	34.8±6.0	0.6559
Hemoglobin (g/L)	81.0±16.5	79.2±16.9	0.2846
24 h urine volume (mL)	1156.0±647.4	1154.0±579.1	0.9977
eGFR (%)	5.0±1.9	5.3±2.5	0.7843

### Operation-related parameters

The success rate of surgery, operative costs and analgesic use were not significantly different. However, the “Half-Perc” group showed significantly shorter operative time, shorter incision length, less bleeding volume and a less postoperative pain score. It is worth to note that the delay in the start of PD program was significantly shorter in the “Half-Perc” group (Table 2).

**Table 2:** Operation-related parameters

Operative parameters	“Half-Perc”	Open surgery	*P* value
Patient (n)	126	114	
Operative time (mins)	53.2±25.2	86.6±31.4	< 0.0001
Incision length (cm)	3.3±0.9	4.1±0.4	< 0.0001
Bleeding volume (mL)	8.0±5.6	16.0±17.3	< 0.0001
Postoperative pain score within 24 hours	3.2±2.2	4.5±2.2	0.0021
Use of anesthetic within 24 hours	13 (11.4%)	21 (16.7%)	0.2429
Success rate of surgery	124 (98.4%)	113 (99.1%)	0.6210
Operative cost (CN¥)	1709.0±43.4	1728.0±67.3	0.0024
delay in start of PD [median (interquartile range) days]	3 (2.8-5.0)	5 (3.0-6.3)	< 0.0001

### Mechanical complications

During the 1-year of follow-up period, 17 episodes of mechanical complications associated with initial PD catheters were observed (Table 3). Catheter dysfunction was the most important mechanical complication that occurred in 6 (2.5%) patients. There were also 3 episodes of dialysate leakage, 4 episodes of hemoperitoneum, 1 episode of outer cuff extrusion and 1 episode of inflow or outflow pain. There were no other significant differences in mechanical complications when comparing the two groups.

**Table 3:** Mechanical complications after one-year follow-up

Complications	“Half-Perc”	Open surgery	*P* value
Patient (n)	126	114	
Catheter malfunction	5 (4.0%)	1 (0.9%)	0.1024
Migration	2 (1.6%)	0 (0.0%)	
Non-migration	3 (2.4%)	1 (0.9%)	
Dialysate leak	2 (1.6%)	1 (0.9%)	0.6210
Pleural	1 (0.8%)	0 (0.0%)	
Scrotal	1 (0.8%)	1 (0.9%)	
Hemoperitoneum	2 (1.6%)	2 (1.8%)	0.9196
Outer cuff extrusion	0 (0.0%)	1 (0.9%)	0.2921
Inflow/outflow pain	1 (0.8%)	0 (0.0%)	0.3405

### Clinical outcomes

**Fig. 3:**
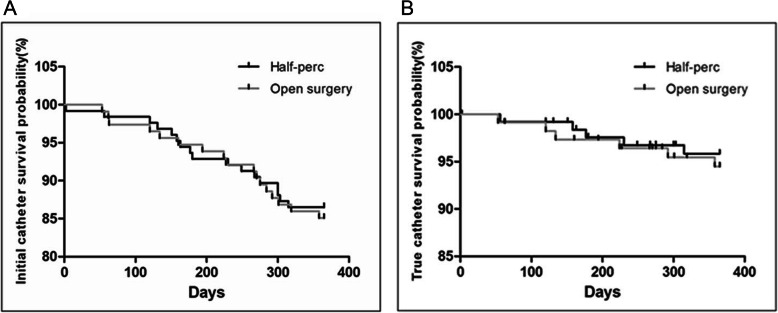
Kaplan–Meier plot for catheter survival based on PD catheter placement technique. A. Initial catheter survival. Log-rank (Mantel-Cox) Test:0.08903; Hazard Ratio:0.9026; 95% CI of ratio:0.4603 to 1.770. B. True catheter survival. Log-rank (Mantel-Cox) Test:0.2232; Hazard Ratio:0.7518; 95% CI of ratio:0.2302 to 2.455. Survival did not significantly differ between the “Half-Perc” technique and open surgery groups

There were 5 episodes of peritonitis in the “Half-Perc” group and 11 of these complications in the open surgery group for an overall rate of 0.0286 episodes per patient–year. Episodes of exit-site infection numbered 4 for a rate of 0.0078 episodes per patient–year. The peritonitis, exit-site infection, overall patient mortality and catheter removal rate did not statistically differ by the two groups (Table 4). There was no surgical mortality that occurred in either group. The two groups did not significantly differ in reasons behind patient mortality or catheter removal (Table 5). The Kaplan-Meier plot of the initial catheter survival also did not statistically differ between the “Half-Perc” technique versus open surgery (Fig. 3a). Excluding deaths unrelated to catheter, renal recovery and renal transplantation (accounting for 12 patients in the “Half-Perc” group and 11 patients in the open surgery group), the Kaplan-Meier plot of the true catheter survival rate also showed no significant differences for the two groups (Fig. 3b).

**Table 4:** Clinical outcomes after one-year follow-up

Clinical outcomes	“Half-Perc”	Open surgery	*P* value
Patient (n)	126	114	
Peritonitis			
Episodes	5 (4%)	11 (9.6%)	0.0781
Episodes/patient–year	0.0184	0.0399	< 0.0001
Exit-site infection			
Episodes	1 (0.8%)	3 (2.6%)	0.2667
Episodes/patient–year	0.0001	0.0285	< 0.0001
Initial catheter survival	109 (86.5%)	97 (85.1%)	0.7527
True catheter survival	121 (96.0%)	108 (94.7%)	0.6319
Patient mortality	4 (3.2%)	2 (1.8%)	0.4816
Catheter removal	13 (10.3%)	15 (13.2%)	0.4937

**Table 5:** Reason for patient mortality and catheter removal

Reason	“Half-Perc” (*n* = 126)	Open surgery (*n* = 114)	*P* value
Total number of subjects	17 (13.5%)	17 (14.9%)	0.7527
**Overall patient mortality**	4 (3.2%)	2 (1.8%)	0.4816
Catheter-related Peritonitis	1 (0.8%)	0 (0.0%)	
Catheter-unrelated Pneumonia	0 (0.0%)	1 (0.9%)	
Myocardial infarction	1 (0.8%)	0 (0.0%)	
Cardiac failure	1 (0.8%)	0 (0.0%)	
Stroke	0 (0.0%)	1 (0.9%)	
Unknown	1 (0.8%)	0 (0.0%)	
**Overall catheter removal**	13 (10.3%)	15 (13.2%)	0.4937
**Catheter-unrelated**	9 (7.1%)	9 (7.9%)	0.8252
Resolution of renal failure	1 (0.8%)	1 (0.9%)	
Voluntary change to hemodialysis	2 (1.6%)	0 (0.0%)	
Renal transplantation	6 (4.7%)	8 (7.0%)	
**Catheter-related**	4 (3.2%)	6 (5.2%)	0.4188
Dialysate leak	1 (0.8%)	0 (0.0%)	
Hernia	1 (0.8%)	0 (0.0%)	
Peritonitis	0 (0.0%)	3 (2.6%)	
Inadequate solute clearance	2 (1.6%)	3 (2.6%)

## Discussion

In this study, we aimed to evaluate the effects of the “Half-Perc” technique versus the open surgery technique in PD catheter placement. The major finding was that the “Half-Perc” placement of PD catheters was non-inferior to a traditional open surgery. This novel technique was characterized by a less invasive injury and shorter delay in the start of the PD program compared to traditional open surgery. After the 365-day follow-up, overall common complications and clinical outcomes did not statistically differ by the two groups.

One of our most recent studies used a modified half-percutaneous (“Half-Perc”) technique for PD catheter placement [10]. The “Half-Perc” technique is based on a special metal trocar similar to the Tenckhoff trocar and a guide wire. This novel percutaneous technique shares some of the benefits of both the Seldinger percutaneous technique and traditional open surgery including minimal tissue trauma, quick postoperative recovery, purse-string suture and relatively low incidence of catheter migration and dialysate leakage [13]. Even though this novel technique is a safe, effective and reliable method for catheter placement during the perioperative period [10], the long-term clinical effects for catheter insertion are not clear and whether this novel method is superior to others remains unknown.

Individuals diagnosed with ESRD that were ≥ 18 years of age and who were undergoing first PD catheter placement in our center were eligible candidates for this study. The exclusion criteria of patients included: (1) severe obesity [body mass index (BMI) > 35] (open surgical group), (2) previous abdominal trauma, surgery or a history consistent with adhesions (patients with a history of appendectomy, nephrectomy, cholecystectomy and cesarean section were included in the study) (“Half-Perc” group), (3) a history of serious diseases of the lung, heart or other organs, (4) severe medical comorbidity including bleeding diatheses and abnormalities of coagulation tests, (5) severe psychiatric disease, (6) issues living independently. Even though the percutaneous technique is not commonly recommended for inserting PD catheters in patients who received previous abdominal surgeries [8, 16], the novel “Half-Perc” technique for catheter placement is generally safe [10, 13]. In this study, results revealed no intestinal perforation risk. The high incidence of malposition was not observed by “Half-Perc” even though this technique is “blind” without direct visualization of the peritoneum. We postulate that a guide wire helps the catheter reach an ideal position in the abdominal cavity through the opening of the peritoneal membrane and the empty sensation with the modified metal trocar contributes greatly to protective effects in intra-abdominal visceral damage.

PD catheter implantation often leads to several catheter-associated complications. Bleeding is a common complication and severe bleeding was observed in 1–5% of procedures [17]. In this study, intraoperative bleeding was observed less in “Half-Perc” group compared to the open surgical group and only 2 (1.6%) patients showed abdominal bleeding in “Half-Perc” group at 2 weeks and 6 months after surgery, respectively. There are diverse results in the literature regarding the incidence of dialysate leakage, likely due to various methods inserting PD catheters and different follow-up periods. Previous work shows that the occurrence rate of dialysate leakage is as high as 12.8% after catheter insertion, particularly within the first few weeks patients commence regular PD program [18]. In this study, only 2 (1.6%) cases of dialysate leakage occurred after using the “Half-Perc” technique, which where pleural leakage was observed 2 months after surgery and another where scrotal leakage was observed 3 months after surgery.

Catheter dysfunction, most commonly seen in PD treatment modality, is a major cause of PD technique failure [19, 20] that can result from catheter migration, omental wrapping, blood clots, fibrin or obstruction secondary to infection [21]. Here, catheter dysfunction occurred in five patients (4%) in the “Half-Perc” group and in one patient (0.9%) in open surgical group after commencing PD. The “Half-Perc” group appeared to have a higher rate of catheter migration than the open surgery group within a short-term postoperative follow-up period. However, catheter dysfunction in all patients was corrected through conservative treatments, except for one patient that needed surgical treatment 10 days after PD due to omental wrapping. We further explored the causes of catheter dysfunction and speculated that an earlier treatment may contribute to the high rate of catheter malfunction since the “Half-Perc” group (3 days) showed a shorter delay in the start PD compared to the open surgery group (5 days) after PD catheter placement. We then instructed patients to reduce frequent physical activity after catheter insertion and performed the PD treatment in the patient bed, ultimately eliminating the chance of catheter malfunction.

PD-related infections including peritonitis and exit-site/tunnel infections remain major complication that can lead to technical failure or even death [22]. There are many studies suggesting that the prevention of peritonitis is necessary for the improvement of catheter survival as well as the long-term success of peritoneal dialysis [23]. Overall, we observed less episodes/patients-year of peritonitis and exit-site infection in the “Half-Perc” group during 365 days follow-up time, but both complications were not statistically different. However, it should also be noted that PD catheter-related infections may have non-surgical effects. Therefore, incidence should be shown within 30 days based on recent PD access guidelines [24]. In this study, there were only three patients experiencing peritonitis within 30 days after catheter insertion in open surgical group, and only one exit-site infection was observed in the “Half-Perc” group.

The regular PD program was usually started at 7 days after catheter insertion [25, 26]. However, the timing of PD catheter implantation was relatively late and the initiation of PD was early in China since most ESRD patients needed urgent dialysis treatment. Thus, the average duration of delay in the start of the regular PD program after PD catheter placement was 3 days (interquartile range, 2.8–5 days) in the “Half-Perc” group and 5 days (interquartile range, 3–6.3 days) in the open surgery group. It is worth mentioning that the “Half-Perc” technique may be helpful for critically ill patients with acute kidney injury or chronic kidney disease in the late stages of uraemia or critical cases beyond kidney disease such as acute necrotic hemorrhagic pancreatitis [27] since urgent-start PD treatment can be immediately performed after catheter placement due to the use of local anesthesia and minimal tissue damage during the catheterization procedure.

## Conclusions

The “Half-Perc” technique may be a suitable alternative to achieve the same clinical efficacy for PD catheter placement in ESRD patients, and it carries minimal invasiveness and allows for a quick start of the PD program. However, this new technique still needs to be confirmed by prospective cohort studies or randomized controlled trials.

## Data Availability

The datasets used and/or analyzed during the current study are available from the corresponding author on reasonable request.

## Abbreviations

“Half-Perc”: Half percutaneous
ESRD: End-stage renal disease
PD: Peritoneal dialysis
HD: Hemodialysis
BMI: Body mass index
MAP: Mean arterial pressure
Scr: Serum creatinine
BUN: Blood urea nitrogen
eGFR: Estimated glomerular filtration rate
SD: Standard deviation
CI: Confidence interval
HR: Hazard ratio

## References

[1] Yu X, Yang X (2015). Peritoneal dialysis in China: meeting the challenge of chronic kidney failure. Am J Kidney Dis.

[2] Ash SR (2003). Chronic peritoneal dialysis catheters: overview of design, placement, and removal procedures. Semin Dial.

[3] Shahbazi N, McCormick BB (2011). Peritoneal dialysis catheter insertion strategies and maintenance of catheter function. Semin Nephrol.

[4] Crabtree JH, Chow KM (2017). Peritoneal Dialysis catheter insertion. Semin Nephrol.

[5] Chow KM, Szeto CC (2010). Open surgical insertion of tenckhoff catheters for peritoneal dialysis. Perit Dial Int.

[6] Al Azzi Y, Zeldis E, Nadkarni GN, Schanzer H, Uribarri J (2016). Outcomes of dialysis catheters placed by the Y-TEC peritoneoscopic technique: a single-center surgical experience. Clin Kidney J.

[7] Crabtree JH, Burchette RJ (2009). Effective use of laparoscopy for long-term peritoneal dialysis access. Am J Surg.

[8] Peppelenbosch A, van Kuijk WH, Bouvy ND, van der Sande FM, Tordoir JH (2008). Peritoneal dialysis catheter placement technique and complications. NDT Plus.

[9] Nic V, Van BW, Raymond V, Norbert L. Peritoneal dialysis catheters: the beauty of simplicity or the glamour of technicality? Percutaneous vs surgical placement. Nephrol Dial Transplant. 2002;17(2):210–12. 10.1093/ndt/17.2.210 11812867

[10] Peng Y, Zhang D, Zheng T (2019). A half-percutaneous technique for peritoneal dialysis catheter implantation using a modified trocar: a report of 84 cases. Int Urol Nephrol.

[11] Zhu B, Liu L, Hong L, Bo S, Wang Y. CAPD catheter placement technique using a self-developed trocar: 38 cases report. Chin J Blood Purification. 2002;1(3):51–1. 用自行研制的腹腔套管针进行CAPD植管术38例.

[12] Zhu B, Zhu X, Yao X (2016). Peritoneal dialysis catheter insertion technique using a trocar. Chin J Integr Tradit West Nephrol.

[13] Zhao S, Zhu X, Li Q (2019). A simplified percutaneous technique for inserting Tenckhoff catheters for peritoneal dialysis. Clin Nephrol.

[14] Georgiades CS, Geschwind JF (2002). Percutaneous peritoneal dialysis catheter placement for the management of end-stage renal disease: technique and comparison with the surgical approach. Tech Vasc Interv Radiol.

[15] Sanderson MC, Swartzendruber DJ, Fenoglio ME, Moore JT, Haun WE (1990). Surgical complications of continuous ambulatory peritoneal dialysis. Am J Surg.

[16] Crabtree JH (2010). Who should place peritoneal dialysis catheters. Perit Dial Int.

[17] Mital S, Fried LF, Piraino B (2004). Bleeding complications associated with peritoneal dialysis catheter insertion. Perit Dial Int.

[18] Haggerty S, Roth S, Walsh D (2014). Guidelines for laparoscopic peritoneal dialysis access surgery. Surg Endosc.

[19] Tiong HY, Poh J, Sunderaraj K, Wu YJ, Consigliere DT (2006). Surgical complications of Tenckhoff catheters used in continuous ambulatory peritoneal dialysis. Singap Med J.

[20] Blake PG (2008). The critical importance of good peritoneal catheter placement. Perit Dial Int.

[21] Crabtree JH (2015). Peritoneal dialysis catheter implantation: avoiding problems and optimizing outcomes. Semin Dial.

[22] Htay H, Johnson DW (2019). Catheter type, placement, and insertion techniques for preventing catheter-related infections in maintenance peritoneal Dialysis patients: summary of a Cochrane review. Am J Kidney Dis.

[23] Szeto CC, Li PK (2019). Peritoneal Dialysis-associated peritonitis. Clin J Am Soc Nephrol.

[24] Crabtree JH, Shrestha BM, Chow KM (2019). Creating and maintaining optimal peritoneal Dialysis access in the adult patient: 2019 update. Perit Dial Int.

[25] Jwo SC, Chen KS, Lee CC, Chen HY (2010). Prospective randomized study for comparison of open surgery with laparoscopic-assisted placement of Tenckhoff peritoneal dialysis catheter--a single center experience and literature review. J Surg Res.

[26] Jiang C, Xu L, Chen Y, Yan X, Sun C, Zhang M (2014). A modified open surgery technique for peritoneal dialysis catheter placement decreases catheter malfunction. Perit Dial Int.

[27] Li Q, Zhu B, Zhu X (2016). Treatment of necrotizing acute pancreatitis with peritoneal lavage and dialysis by a new simplified technique insert catheters: one retrospective study. Medicine (Baltimore).

